# A novel parameter dense three-dimensional convolution residual network method and its application in classroom teaching

**DOI:** 10.3389/fnins.2024.1482735

**Published:** 2024-10-11

**Authors:** Xuan Li, Ting Yang, Ming Tang, Pengwen Xiong

**Affiliations:** ^1^School of Foreign Language, Shangrao Normal University, Shangrao, China; ^2^School of Advanced Manufacturing, Nanchang University, Nanchang, China

**Keywords:** three-dimensional convolutional neural network, residual network, video sequence, SSD algorithm, behavior recognition

## Abstract

**Introduction:**

Improving the rationality and accuracy of classroom quality analysis is crucial in modern education. Traditional methods, such as questionnaires and manual recordings, are resource-intensive and subjective, leading to inconsistent results. As a solution, computer vision (CV) technologies have emerged as powerful tools for real-time classroom monitoring. This study proposes a novel Dense 3D Convolutional Residual Network (D3DCNN_ResNet) to recognize students’ expressions and behaviors in English classrooms.

**Methods:**

The proposed method combines Single Shot Multibox Detector (SSD) for target detection with an improved D3DCNN_ResNet model. The network applies 3D convolution in both spatial and temporal domains, with shortcut connections from residual blocks to increase network depth. Dense connections are introduced to enhance the flow of high- and low-level features. The model was tested on two datasets: the CK+ dataset for expression recognition and the KTH dataset for behavior recognition.

**Results and Discussion:**

The experiments show that the proposed method is highly efficient in optimizing model training and improving recognition accuracy. On the CK+ dataset, the model achieved an expression recognition accuracy of 97.94%, while on the KTH dataset, the behavior recognition accuracy reached 98.86%. The combination of residual blocks and dense connections reduced feature redundancy and improved gradient flow, leading to better model performance. The results demonstrate that the D3DCNN_ResNet is well-suited for classroom quality analysis and has the potential to enhance teaching strategies by providing real-time feedback on student engagement.

## 1 Introduction

Improving the rationality and accuracy of classroom quality analysis is particularly important for classroom teaching (Xun, 2022). In recent years, many forms of classroom quality analysis have also been studied, mainly including questionnaire form, physiological embodiment method, computer vision (CV) and other methods. At present, the vast majority of classrooms analyze and evaluate the quality of classroom teaching through specialist manual records, after-school questionnaires and other methods. These methods not only consume a lot of human and material resources, but also have a certain subjectivity, so it is unrealistic to get reasonable and accurate results. CV methods could be combined with intelligent equipment with video recording to monitor the students’ class status in real time, study and analyze the students’ facial expression and posture in the classroom, then analyze the classroom quality (Wu et al., 2023). Since the 21st century, it has promoted the development of integrating computer emotion analysis and education and teaching application.

Through the analysis of intelligent video equipment and expression recognition, it can provide teachers with real-time and reliable feedback information in time, facilitate teachers to modify teaching contents, control teaching progress, select teaching methods and adjust teaching difficulties, and greatly promote the change of teaching mode and the improvement of teaching quality. In the field of computer vision, object recognition from video and pictures has always been a hot topic. How to extract robust and representative features is a challenging task (Hong, 2022). For static image recognition, only static features in one image need to be extracted for learning (Niu et al., 2022). For dynamic video recognition, we need to consider not only the relationship between adjacent pixels in a single frame image in the spatial domain, but also the interaction between multiple adjacent frames in the temporal domain (Wanshu and Bin, 2022). At present, the research around video recognition mainly focuses on feature extraction and classifier selection.

For video object classification, the quality of feature extraction is very important, which directly affects the classification effect. The judgment of its quality lies in whether it has high recognition degree, strong robustness, more complete recognition information and so on. The existing feature extraction methods are mainly divided into two categories: local feature methods and global feature methods. The local feature method is to extract the local sub regions or interest points in the video or image. For example, Bengio et al. (1994) proposed gradient based learning algorithms as the duration of the dependencies to be captures increased. Laptev and Lindeberg (2005) first detected multiple spatiotemporal interest points from the video, then built a spatiotemporal cube centered on the interest points and extracted hog and Hof features to represent the motion information. Gers et al. (2002) found that LSTM can learn the subtle differences between spike sequences with intervals of 49 or 50 times steps without the help of any short training samples by connecting the “peephole connections” from its internal cells to its multiplication gate. Ma et al. (2015) proposed a new Bayesian matrix decomposition method for bounded support data. The beta distribution has two parameters, while the two parameter matrices with only non-negative values can be obtained. To provide low rank matrix factorization, non-negative matrix factorization (NMF) technique is applied. Gu et al. (2012) performed Gabor transform on the image, then jointly encoded it by radial network, and realized global classification by cascading multiple classifiers. Although the above methods can well represent the image edge information, its feature dimension is too high and the amount of calculation is too large.

According to the analysis, each feature extraction method has its advantages and disadvantages. In order to make up for each other’s shortcomings and overcome the problems of insufficient description of image information by a single feature and weak robustness, most of the current research methods use mixed features. For example, Liu et al. (2011) proposed a novel method to extract expression features by combining Gabor multi-directional feature fusion and block histogram statistics based on the weak ability of Gabor features to represent global features. Ren et al. (2017) introduced a regional recommendation network (RPN) that shared full image convolutional features with the detection network to achieve almost cost-free regional recommendations. Graves et al. (2005) used convolution neural network and long-term and short-term memory neural network to extract features, and then weighted and fused the extracted features to improve the accuracy and generalization of the model. Ji et al. (2013) developed a 3D CNN model which extracted features from both spatial and temporal dimension for action recognition.

Through the study of the above methods, in order to not only overcome the problem that a single feature does not adequately describe the image information but also simplify the model structure and improve recognition accuracy, this paper proposes a new parallel Dense 3D convolution residual network (D3DCNN_ResNet) method for students’ expression and behavior recognition in English classrooms. This method first uses the single shot multibox detector (SSD) to extract the moving area to be recognized for clipping and preprocessing, and then inputs the crop and the original frame picture into the improved convolution residual network to extract features. The crop can extract local detailed feature information. On the other hand, the original frame can obtain the global overall information, supplement the edge contour features that the former failed to extract completely, and then carry out weighted fusion to improve the robustness and generalization of the model. Due to the large number of parameters and low computational efficiency of the 3D convolutional neural network, a Pseudo 3D convolutional neural network (P3D) (Qiu et al., 2017) is proposed to decompose 3D into (2D + 1D) forms. Pseudo 3D convolution (P3D) decomposes 3D convolution into 2D spatial and 1D temporal convolutions, reducing computational complexity while maintaining performance. It has been applied in video action recognition and medical imaging, balancing accuracy and efficiency. According to the four structures designed in Gu et al. (2012), 2D spatial convolution and 1D temporal convolution are proved to be the most effective.

**Figure 1:**
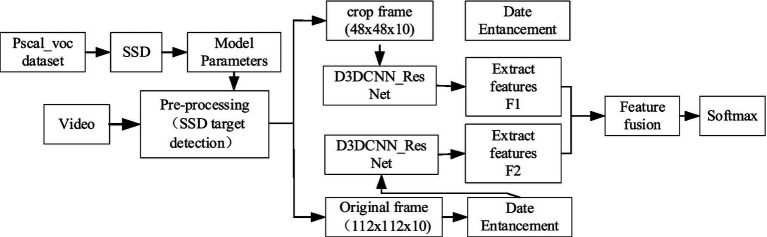
Model structure diagram.

Main contributions:

1. With our proposed D3DCNN_ResNet, a new English classroom quality analysis with low and controllable cost, high precision and reliability combined with facial expression recognition technology is proposed.
2. In the proposed D3DCNN_ResNet, the residual module is added to learn the residual mapping to further solve the problems of gradient descent and over fitting with the deepening of the network depth, and more subtle multi-level features are obtained through the interconnection between different convolution layers and different residual blocks in the residual block.
3. The combination of residual blocks and dense connections not only reduces feature redundancy and improves the gradient correlation of the network, but also reduces the network parameters to a certain extent. Our architecture combines DenseNet and ResNet principles, improving gradient flow and reducing the risk of vanishing gradients in deeper networks. DenseNet’s feature reuse and ResNet’s residual connections have been validated in tasks like object detection and image classification, enhancing feature learning (see Figure 1).

The rest of this letter is organized as follows. In section 2, we present the basic principle and framework of our proposed D3DCNN_ResNet in detail. Section 3 gives the model optimization. Section 4 presents the results and analysis based on the experiment. Finally, the conclusions are given in section 5.

## 2 New parallel D3DCNN_ResNet identification method

### 2.1 Introduction to identification method

This method mainly includes two parts, SSD target detection and improved D3DCNN_ResNet. It can accurately locate the recognition area of the object by using SSD target detection, and get detailed local features through crop, and then use the original frame as a feature supplement to obtain more abundant, complete and robust feature information. SSD target detection is a preprocessing module, and could be used to cut the recognition area corresponding to the entire video sequence. The obtained sequence frames are directly input into the improved D3DCNN_ResNet to extracts features. Since deep learning can combine low-level features into high-level ones through the construction of multiple hidden layers and autonomously learned features, the improved D3DCNN_ResNet model connects different residual blocks and convolution layers within the residual blocks. It fully combines the bottom features with the high-level features, and enhances the flow of feature information in the network. Then, the obtained local and global multi features are fused to better represent the subtle feature information. This structure not only solves the lack of time-domain information extraction in traditional deep learning, but also solves the problems of large parameters and over fitting through the decomposition of the three-dimensional convolution, which improves the recognition rate of the model. The specific implementation steps of the algorithm are as follows:

1. Firstly, SSD target detection algorithm is applied to the input video frame, and the object recognition region is extracted from each frame of the video for clipping preprocessing, which is called the crop.
2. Then, the original frame and the crop are input to the improved D3DCNN_ResNet respectively. The extracted features in RESNET are marked as F1 and F2: F1 is the local detailed feature of the recognition object, and F2 is the global overall feature, which mainly supplements the edge contour information. F1 and F2 also include the fusion between low-level features and high-level features, with feature dimensions of 256.
3. Finally, the original frame features and the crop frame features are fused, encoding the video information. Since the feature level fusion at the full connection layer will greatly increase the parameters of the model, the decision level fusion method is selected in this paper, as shown in Equation 1:

(1) $$R\left(x\right)=\overset{2}{\underset{n=1}{\sum }}W_{n}\times P_{n}\left(x\right)$$

where, *P_n_*(*x*), (0 < *p* < 1) is the output probability value of F1 and F2 at softmax layer, *W* is the weight parameter, which is obtained from the least square estimation of the minimization loss function:


(2) $${\left\{w_{n}\right\}}_{n=1}^{2}=\underset{w_{1},w_{2}}{\arg\min}\left(o-\overset{2}{\underset{n=1}{\sum }}w_{n}\times P_{n}{\left(x\right)}_{F}^{2}\right)$$

The final fusion feature is input into softmax classifier to realize classification and recognition.

### 2.2 Introduction to improved D3DCNN_ResNet

**Figure 2:**
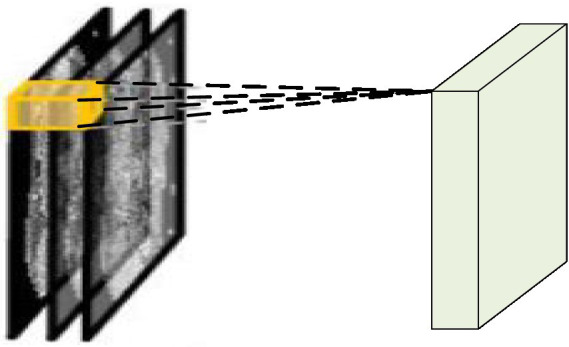
3DCNN schematic diagram.

In video analysis, considering the motion information between consecutive frames, D3DCNN_ResNet is used to stack multiple consecutive frame images to form a cube, and then use 3D convolution kernel to convolute in the cube. As shown in Figure 2, the characteristic value of a certain position of a convolution map is obtained by convoluting the local receptive field of the same position of three consecutive frames on the upper layer. Its advantage is that it can extract the spatial-temporal features at one time and capture the action information of multiple frames in the video sequence.

In the convolution process, on each characteristic graph of any single layer, the value of position (*a*, *b*, *c*) is given by Equation 3:


(3) $$v^{abc}=\tanh\left(t\overset{H-1}{\underset{h=0}{\sum }}\overset{W-1}{\underset{w=0}{\sum }}\overset{D-1}{\underset{d=0}{\sum }}xy^{\left(a+h\right)\left(b+w\right)\left(c+d\right)}+z\right)$$

where tanh () is a hyperbolic tangent function, index *T* and value *x* are the connection parameters of the current characteristic graph. *h*, *W*, and *D* are the height, width and time dimensions of the three-dimensional convolution kernel, and *z* is the deviation of the characteristic graph.

**Figure 3:**
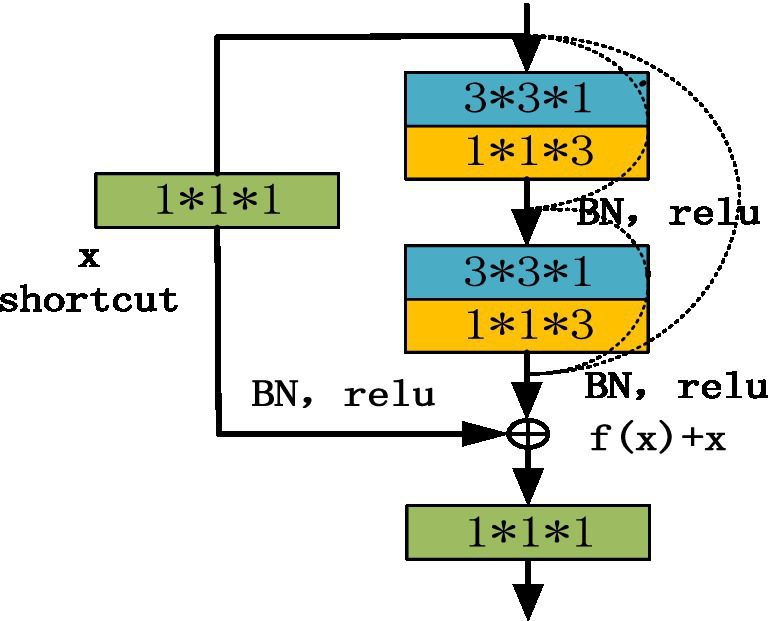
Schematic diagram of residuals module.

**Figure 4:**
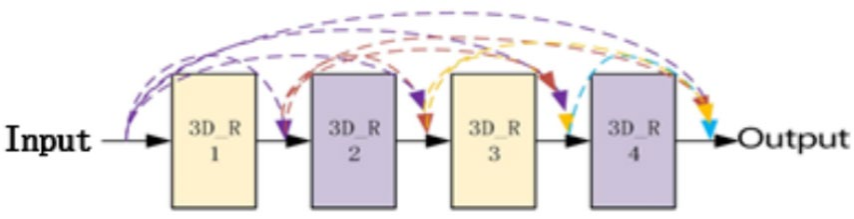
Dense connection diagram of 3DCNN RESNET.

The deep 3DCNN is more effective in feature extraction, but 3DCNN has too many parameters. After increasing the depth, it will further affect its learning efficiency and accuracy, and cause the gradient to disappear. Therefore, 3D is decomposed into (2D + 1D) structure, which means (*v*1*v2*v3) the filter is replaced by (*v*1*v2*1) and (1*1*v3) serially, and samples in the spatial and temporal regions respectively, effectively reducing the amount of parameters. At the same time, residual blocks are added to 3DCNN to simplify the training of deep network. Formally, the required bottom mapping is expressed as *h* (*x*), that is, the optimal solution mapping after the input sample *x*. Let the superimposed nonlinear layer fit the residual mapping of *F* (*x*) = *h* (*x*) − *x*, and convert the original mapping to Equation 4.


(4) $$y=f\left(x,w\right)+x$$

where *x* is the output value of the upper neural unit, *w* is the weight of the neuron, and *y* is the output value of the activation function in the neuron. In addition, the input of each convolution layer in the residual block is composed of the output of all previous convolution layers, and the output of all previous residual blocks is used as the input of the next residual block to improve the gradient correlation of the network, and ResNET can maximize the information flow while reducing network redundancy. The basic structure of the improved D3DCNN_ResNet is shown in Figure 3. In order to convert the two-dimensional residual unit into a three-dimensional structure, it is used for encoding spatiotemporal video information. According to the three-dimensional convolution theorem, the basic residual element is modified as follows:

As shown in Figure 3, the residual block has two parts: quick connection (solid line) and dense connection (dotted line). There is a three-dimensional convolution layer (conv3d) in the shortcut, which is mainly used to change the input dimension and increase its nonlinear representation so as to match the output dimension of the main path in the subsequent addition steps, so that the feedforward/back propagation algorithm can be carried out smoothly. In addition, the three-dimensional convolution unit is replaced by (3*3*1) and (1*1*3) serial. Secondly, dense connection not only makes each three-dimensional convolution unit take the output of the previous three-dimensional convolution unit as the input, but also makes dense connection between each residual block to splice the features. As shown in Figure 4, this structure can deepen the network depth and improve the model representation ability. At the same time, due to the repeated use of convolution features, it can appropriately reduce the number of convolution cores to achieve a certain anti over fitting effect. In addition, 1*1*1 convolution kernel is used at the end of convolution kernel, which aims at feature aggregation and channel adjustment to reduce the amount of calculation. The comparison results are as follows (taking the spatial size as an example): after the first residual convolution, the characteristic size is 24*24*30 if the number of data channels is not reduced, continuing the convolution (the number of convolution cores is set to 8), and the amount of computation is:


$${\left(24^{*}24^{*}76\right)}^{*}\left(3^{*}3\right)*38+{\left(24^{*}24^{*}38\right)}^{*}\left(3^{*}3\right)*30\approx 20.88\;\mathrm{million}$$

If 1*1*1 convolution is used to compress the channel information so that the feature size is 24*24*16, the amount of computation is:


$$\begin{array}{l} {\left(24^{*}24^{*}48\right)}^{*}\left(3^{*}3\right)*38+{\left(24^{*}24^{*}24\right)}^{*}\left(3^{*}3\right)\\ {}*16+{\left(24^{*}24^{*}16\right)}^{*}\left(1^{*}1\right)*30\approx 8.24\;\mathrm{million} \end{array}$$

Through the above comparison, the calculation amount is reduced by about 1.5 times after adding 1*1*1 convolution.

**Figure 5:**
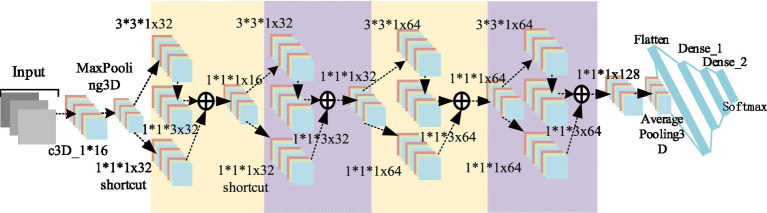
D3 DCNN RESNET structure.

The overall convolution structure of D3DCNN_ResNet used in this paper is shown in Figure 5. The input of the network consists of 10 consecutive frames of images. Take the input crop frame as an example, the spatial size clipping processing of each crop frame image is 48*48, that is, the size of the input video sequence is (10*48*48), and in the input conv3d_1. Before filling, use zeropadding to add dimensions to prevent the loss of image edge information. After filling, the size becomes 50*50. Since the image dimension is high, input conv3d_1 after convolution dimensionality reduction, 16 feature maps with the size of 50*50*10 are obtained, and then downsampling is performed on each feature map in the way of maximum sampling with the size of 1*2*2, so that the number of feature maps is the same and the spatial resolution is reduced. The next layer is to insert the residual blocks of quick connection and dense connection, and get 32 feature maps with the size of 24*24*10. Then, the depth of the joint feature data size is reduced to 16 by the 1*1*1 convolution kernel. In this paper, four residual blocks are used to carry out residual convolution in turn. Finally, 128 characteristic maps with the size of 24*24*10 are obtained. Then, 64 characteristic maps with the size of 12*12*10 are obtained through mean sampling. The data is compressed into one dimension in the flatten layer, and a 256-dimension output feature is obtained through two dense layers. After each convolution layer, the activation function and the batch normalization (BN) layer are connected. Both activation functions use the ReLU function. The input original frame size is 112*112, and the process is the same as that shown above.

### 2.3 SSD target detection

Target detection is a kind of technology that the computer analyzes and distinguishes by extracting the typical features of the target. Its main task is to find out the interested objects in the image and determine their position and size. In this paper, SSD target detector is used to detect human face and human body. Compared with YOLO algorithm, it has better robustness to objects of different scales. Compared with R_CNN series of algorithms, it omits the process of generating candidate boxes, and the calculation speed is faster.

SSD target detection is an end-to-end image target detection method. It directly extracts features from input data to predict object classification and location, which greatly improves the detection speed. The basic network structure of SSD algorithm is VGG16, and the 5-layer network in front of VGG16 is adopted. First, the last two full connection layers are changed into convolution layers by using the atrus algorithm, then three convolution layers and a pooled layer are added for convolution and down sampling processing, and finally two different 3*3 convolution cores are used for convolution, and the detection results are obtained by non-maximum suppression method. For each feature layer, the scale size of the default box is calculated according to the following formula:


(5) $$S_{k}=S_{\text{min}}+\frac{S_{\text{max}}-S_{\text{min}}}{m-1}\left(k-1\right),k\in \left[1,m\right]$$

where, *S*_min_ the value of 0.2 indicates that the scale size of the bottom layer is 0.2; the value is 0.9, indicating that the scale size of the highest layer is 0.9; from the formula, the first layer *S*_min_ = s1, *S*_max_ = s2; the second layer *S*_min_ = s2, *S*_max_ = s3, and so on. *M* represents the number of characteristic layers. The aspect ratio is expressed in AR, and the value is AR = {1, 2, 3, 1/2, 1/3}, then the width and height of each default box are calculated as follows:


(6) $$w_{k}^{a}=S_{k}\sqrt{a_{r}},h_{k}^{a}=\frac{S_{k}}{\sqrt{a_{r}}}$$

The biggest contribution of SSD algorithm is to propose a multi-scale feature layer prediction method. The calculated default box can basically cover the objects of various shapes and sizes in the input image.

**Figure 6:**
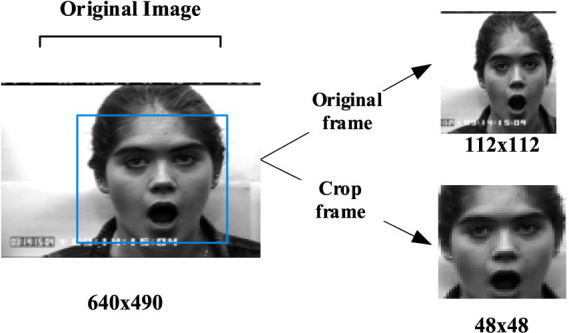
An expression case for clipping. CK+ expression data set is from Lucey et al. (2010).

**Figure 7:**
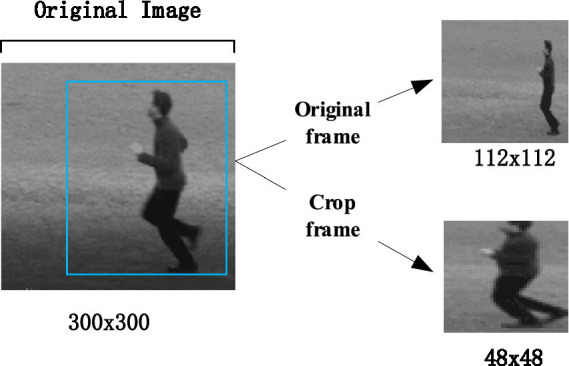
A behavior case for clipping. KTH behavior data set is from Laptev and Lindeberg (2005).

Pascal_voc datasets provide a set of standardized and excellent datasets for image recognition and classification. This article first uses Pascal_voc 2007 and 2012 datasets to get the pre-training model, and then the model file configuration is modified according to their own detection targets to train their own datasets. After the SSD detection, the position of human face and human body in the picture can be determined. Since VOC provides data annotation information, and it does not need to mark the local position manually, so it is faster and more convenient to detect. Then a bounding box is generated for clipping, as shown in Figures 6, 7.

## 3 Model optimization

Model optimization is very important in the training process of neural networks. Making correct decisions in the process of configuring training, verification and testing data sets will help us to create an efficient neural network to a great extent. Selecting appropriate super parameters to train an optimal decision model is the most important link of model optimization.

### 3.1 Activation function and local normalization

**Figure 8:**
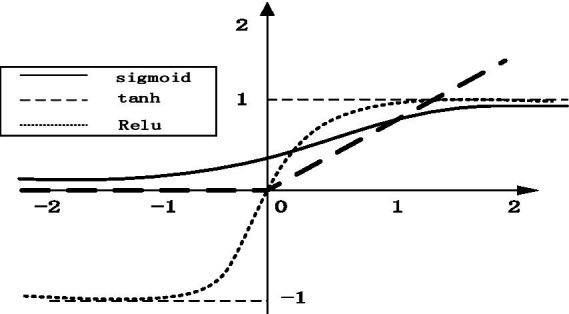
Comparison of three activation functions.

The most important function of activation function is to make the neural network converge and make the operation in the neural network nonlinear so as to construct various valuable functions. Commonly used activation functions include tanh, ReLU, Leaky ReLU, etc. As shown in Figure 8, ReLU is recognized as the best activation function compared with other activation functions, which has the following advantages: (1) simple formula and fast calculation speed; (2) when the input is greater than 0, the gradient is constant to avoid gradient saturation; (3) fast convergence. Therefore, the ReLU activation function is adopted in this paper. The formula is as follows:


(7) $$\mathrm{ReLU}\left(x\right)=\max\left(0,x\right)$$

In order to solve the data distribution change caused by the input data of each layer after updating the parameters, this paper adds batch normalization (BN) (Ioffe and Szegedy, 2015) to the activation function of each layer, and the formula is as follows:


(8) $$y=\frac{\gamma }{\sqrt{Var\left[x\right]+\varepsilon }}\cdot x+\left(\beta -\frac{\gamma E\left[x\right]}{\sqrt{Var\left[x\right]+\varepsilon }}\right)$$

where, *γ* is *β* a learnable reconstruction parameter, so that the network can learn and recover the characteristic distribution to be learned by the original network. *E*[*x*] is the average of the mean values of all samples, and Var[*x*] is an unbiased estimation using the standard deviation of each sample. The calculation process is to calculate the average value and variance of all neurons of a characteristic map corresponding to all samples, and then normalize the neural units of the characteristic map.

### 3.2 Loss function and regularization

The optimization degree of the network model depends on the size of the loss function, which mainly represents the difference between the predicted value and the real value of the model for a specific sample. The loss function used in this paper is the categorical cross entropy loss:


(9) $$C=-\frac{1}{n}\underset{x}{\sum }\left[y\ln a+\left(1-y\right)\ln\left(1-a\right)\right]$$

where *y* is the expected output, a is the actual output of neurons, *n* is the number of input samples in the same batch (batch size), and cross entropy *C* is the distance between the actual output probability and the expected output probability, that is, the smaller *C* is, the closer the two probability distributions are.

In order to prevent the network from over fitting, this paper adds a random dropout regularization term to the loss function of the output layer. When the neural network propagates forward, the activation value of a neuron stops working with a certain probability *P*. *P* is set to 0.5, which makes the model more generalized and less dependent on some local features. At the same time, it reduces the number of training nodes and improves the learning speed of the algorithm.

### 3.3 Optimization algorithm

The optimization algorithm is to optimize the parameters in the network and calculate the gradient of parameters at each layer by back propagation through the error obtained from the loss function. In this paper, rmsprop algorithm is used to update the parameters of the neural network. The iterative update formula is as follows:


(10) $$\begin{array}{l} s_{dw}=\beta s_{dw}+\left(1-\beta \right)dW^{2}\\ s_{db}=\beta s_{db}+\left(1-\beta \right)db^{2}\\ W=W-\alpha \frac{dW}{\sqrt{s_{dw}+\varepsilon }}\\ b=b-\alpha \frac{db}{\sqrt{s_{db}+\varepsilon }} \end{array}$$

From the above formula, *s* is a smoothing of the square of the gradient, and *β* is an index of gradient accumulation, with a typical value of 0.999. When updating the weight *W* and offset *B*, the gradient is divided by $\sqrt{S_{dw}+\varepsilon }$ first, which is equivalent to normalizing the gradient. If the gradient oscillates greatly in one direction, the step size should be reduced. If the oscillation is large, the s in this direction is also large. After division, the normalized gradient becomes smaller; if the gradient oscillation in a certain direction is very small, the normalized gradient becomes larger after division. By using the differential square weighted average for the gradient of weight *W* and offset *B*, it is helpful to eliminate the direction with large swing amplitude, which is used to correct the swing amplitude, so that the swing amplitude of each dimension is small. On the other hand, it also makes the network function converge faster. To prevent the denominator from being zero, a very small value *ϵ* is used for smoothing the general value is 10^−8^.

## 4 Experiment

In order to verify the effectiveness of D3DCNN_ResNet proposed in this paper, the algorithm is applied to expression recognition and behavior recognition. Experimental verification is carried out on CK+ and KTH datasets respectively. The experiment is based on Python keras. The operating system: 64-bit Windows 10 Home Chinese version. CPU: Intel Core i7-6700. Graphics card: Intel HD Graphics 530. Memory: 8GB DDR2.

### 4.1 Data preprocessing

The data set used for expression recognition in this paper is CK+ database (Lucey et al., 2010), which is currently the most widely used database for expression recognition. It contains 593 expression video sequences from 123 people. The CK+ dataset is commonly used for facial expression recognition and contains video sequences where each sequence starts from a neutral expression and peaks at a specific emotion. The seven expressions are happiness, sadness, anger, fear, surprise, disgust, and contempt. The KTH dataset, primarily used for action recognition, consists of six types of human actions (walking, jogging, running, boxing, handwaving, and handclapping) performed under different conditions, providing a robust basis for behavior recognition experiments.

**Figure 9:**
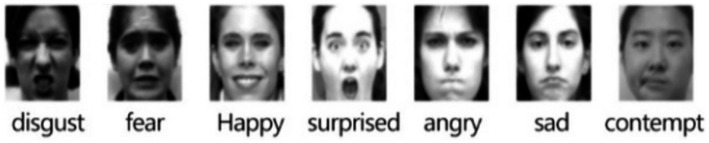
Seven expressions in the CK+ dataset. CK+ expression data set is from Lucey et al. (2010).

As shown in Figure 9, it has seven basic expressions: happiness, sadness, anger, fear, surprise, disgust and contempt.

**Figure 10:**
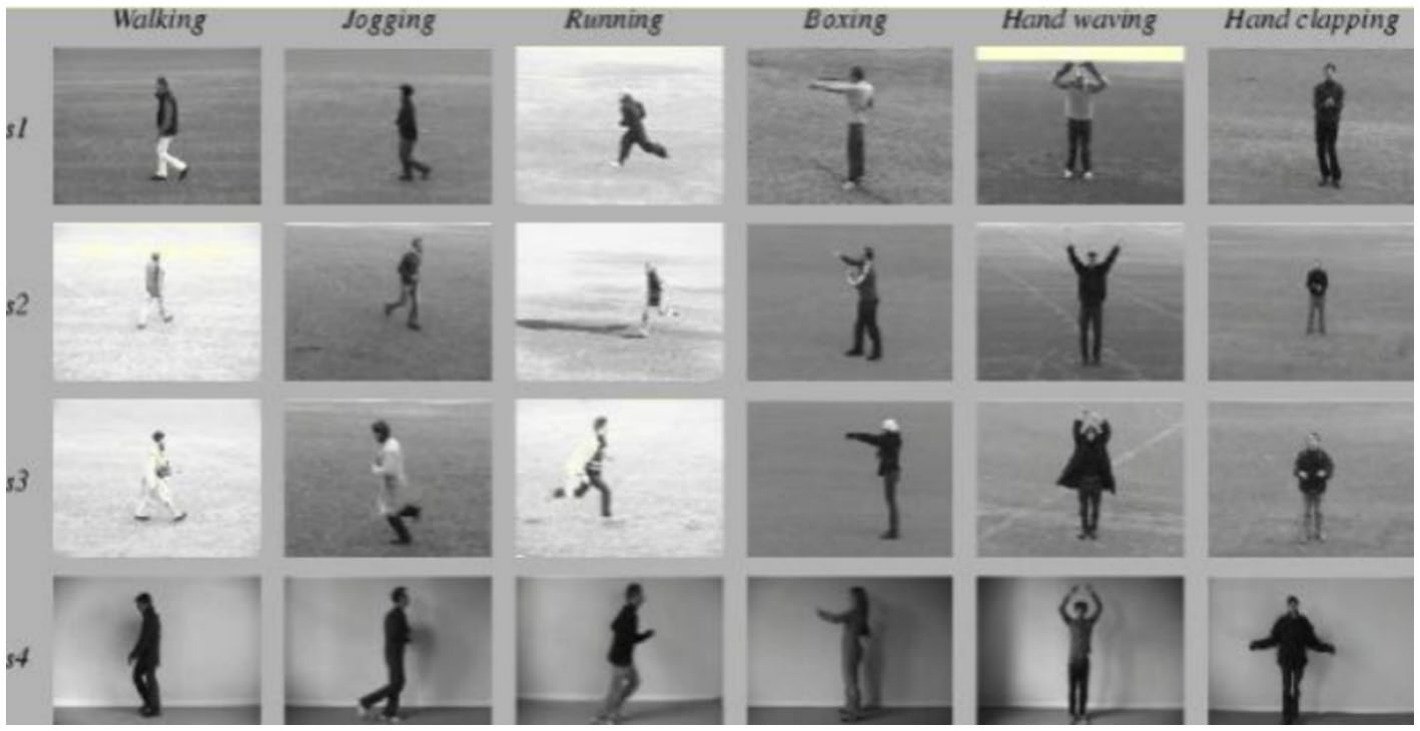
Six behaviors in the KTH dataset. KTH behavior data set is from Laptev and Lindeberg (2005).

In the experiment of behavior recognition, this paper uses KTH data set to verify. The KTH data set is composed of a total of 600 short videos, in which 25 people perform 6 actions under 4 scenarios: “Walking,” “Jogging,” “Running,” “Boxing,” “Handwaving” and “Handclapping,” as shown in Figure 10.

**Figure 11:**
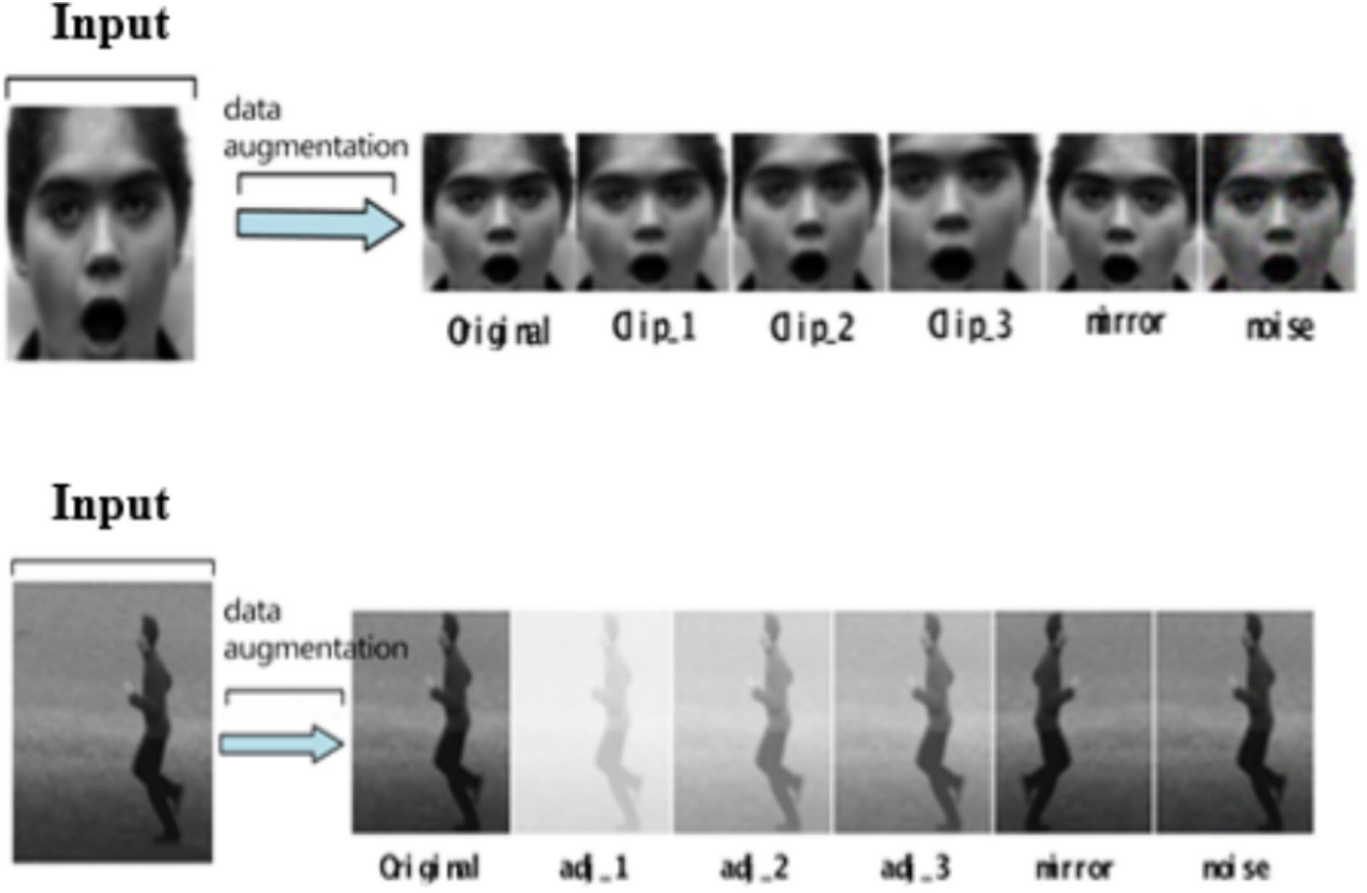
Data augmentation diagram. CK+ expression data set is from Lucey et al. (2010). KTH behavior data set is from Laptev and Lindeberg (2005).

In the experiment, the input original frame size is uniformly specified as 112*112. In both expression recognition and behavior recognition, 10 consecutive frame images are selected as model input. SSD detector is used to detect the face and human body parts and cut them to 48*48 to get the crop. Due to the small number of CK+ and KTH data samples, the existing standard data sets are augmented (such as random clipping, contrast adjustment, noise, mirror image, etc.) to enrich the training data. These augmentation techniques were selected due to their effectiveness in enhancing model robustness by diversifying the training data. Specifically, random cropping helps the model focus on different parts of the image, while contrast adjustment and noise addition improve the model’s ability to generalize to varying lighting conditions and image quality, which are common challenges in real-world classroom environments. As shown in Figure 11, some data augmentation results are shown. Three pieces of CK+ are randomly clipped, one piece of mirror image, one piece of random noise, three pieces of KTH are randomly adjusted for contrast, one piece of mirror image, and one piece of random noise.

The experiment in this paper adopts the method of cross-validation, where the experimental samples are randomly divided into five parts: four are used as the training set, and the other as the test set for final model evaluation. Through training, the accuracy of the four training sets is obtained, and the average value is taken as the accuracy index of the training set in this paper. The accuracy of the test set is used as the accuracy index of the verification set. When training the model, set the batch size to 2, iterate 50 times, and print the results once per iteration.

### 4.2 Experimental results of CK+ expression data set

**Figure 12:**
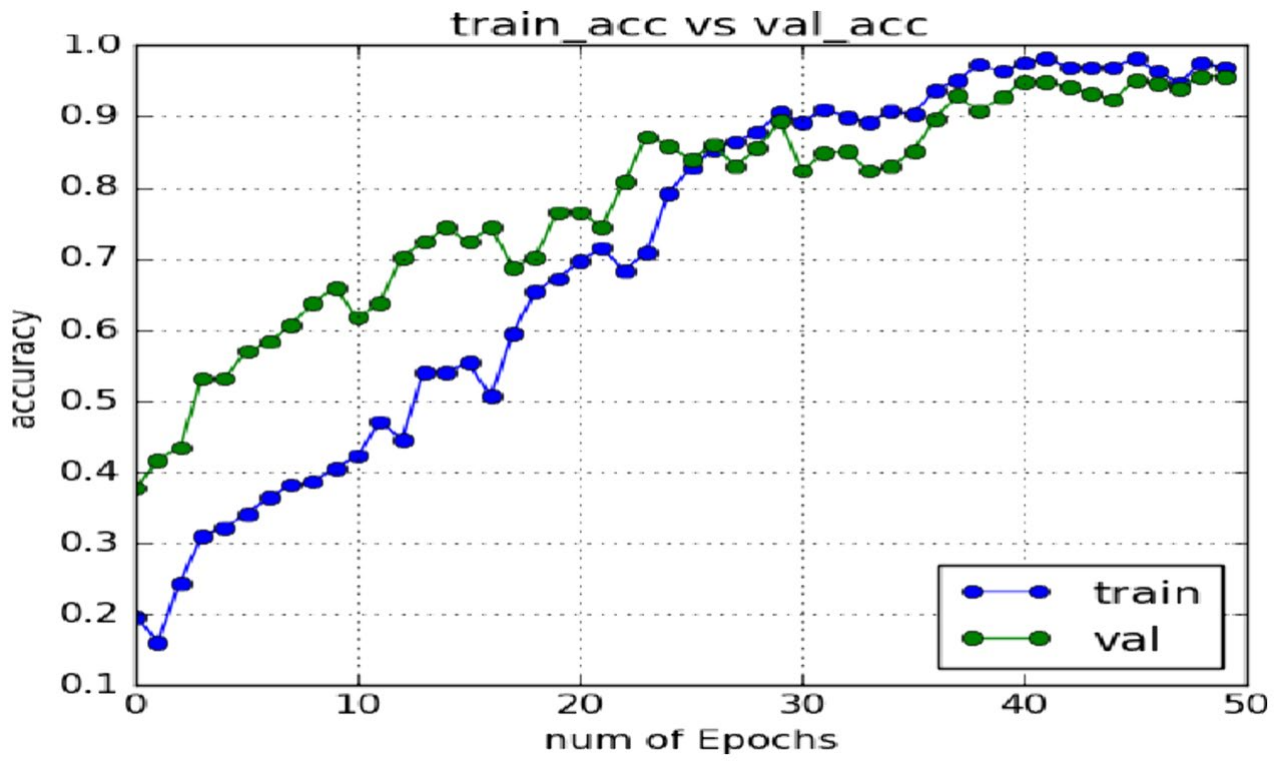
Accuracy of network in training set and test set.

**Figure 13:**
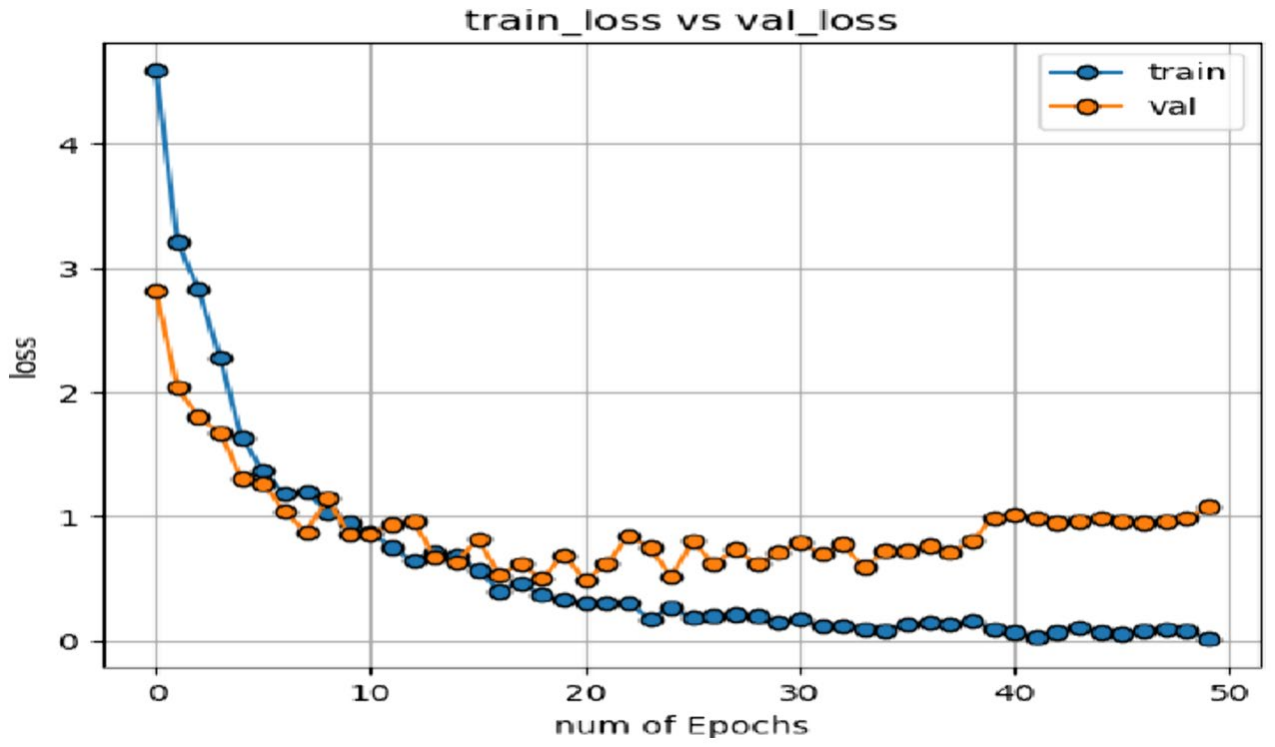
Loss function of network on training set and test set.

**Figure 14:**
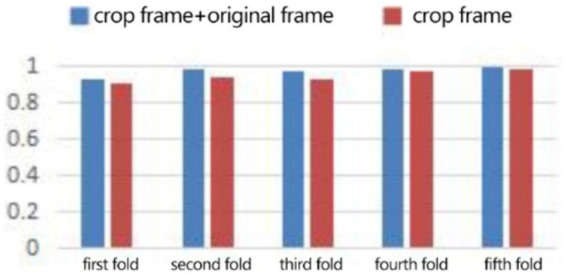
Compare the five-fold recognition rate of crop frame with crop frame + original frame.

**Figure 15:**
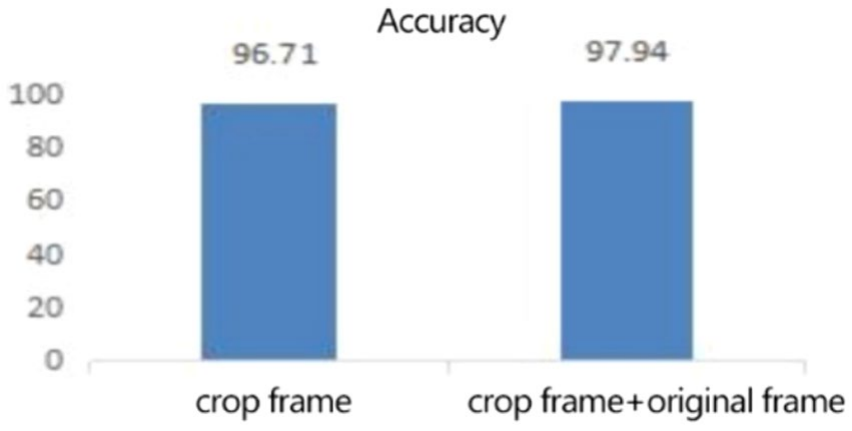
The average recognition rate (%) of clip frames and clip frames + original frames.

In the expression recognition experiment, Figures 12, 13 show the iterative process of the network in the CK+ data set. According to the accuracy curve and loss function curve of the training set and the test set in the training process, the accuracy of the method in this paper is high with the best recognition rate of 97.94%, and the convergence speed of the network is fast. The observed improvements in recognition rates were statistically significant based on standard deviation measures across the five-fold cross-validation. Furthermore, the preprocessing steps, including data augmentation techniques such as random cropping and contrast adjustment, contributed to the robustness of the model, helping to mitigate overfitting and improve generalization. Figure 14 shows the 50% recognition accuracy achieved by using the crop frame alone and by fusing the crop frame with the original frame. Figure 15 shows the corresponding average recognition accuracy. It can be seen from the two figures that the recognition accuracy of the fusion of the two features is high. The effect, improved by 1.23%, is not very significant. The reason is that expression recognition mainly depends on the detail changes of local areas such as eyes, eyebrows and mouth. Therefore, using crop frames alone can achieve better results.

In order to further evaluate the experimental results, it is compared with other frontier methods. Table 1 shows the comparison results of different algorithms on CK+. All comparison experiments were conducted under identical conditions, including the same data preprocessing, model initialization, and hyperparameter settings (e.g., learning rate, batch size). This ensured that the observed differences in performance were solely due to the models’ architectures and not other external factors. Fan and Tjahjadi (2015) fused Phog top and optical flow method to capture changes in facial shape. Yu et al. (2016) introduces a special Bayesian network to capture the time relationship between facial changes, and develops corresponding facial modeling and recognition algorithms to improve the training and recognition speed. Yang et al. (2017) integrates two network models: 3D spatiotemporal network and static network. The former is used to extract spatiotemporal information, and the latter is used to extract the static features of key frames, and then make up for the lack of feature information through model fusion. Wang et al. (2018) proposed an expression recognition method combining dynamic texture information and motion information. Liu et al. (2014) uses 3DCNN to extract local features for fusion to recognize expression. Kacem et al. (2017), firstly, the face is mapped to the Riemannian manifold of positive semi definite matrix, and then the time parameter trajectory is established. Finally, the improved ppfSVM is used for classification, so as to improve the recognition accuracy. In this paper, 3DCNN is used to extract video sequence features, add quick connection to increase network depth, add high-level and low-level features of dense connection, input the clip frame and original frame detected by SSD respectively for training, and fuse the extracted two dense features for classification and recognition. Our proposed D3DCNN_ResNet achieves a recognition rate of 97.94% on CK+ database, which is superior to other methods.

**Table 1:** Comparison results of different algorithms on CK+.

Method	Feature	Recognition rate
Fan and Tjahjadi (2015)	Phog-top and optical flow	90.9
Yu et al. (2016)	LABN	88.1
Yang et al. (2017)	3D model + static model	97.6
Wang et al. (2018)	STWLD and BOHF	91.6
Liu et al. (2014)	3DCNN	85.9
Kacem et al. (2017)	Space–time geometry	96.8
This article	D3DCNN_ResNet	97.9

### 4.3 Experimental results of KTH dataset

**Figure 16:**
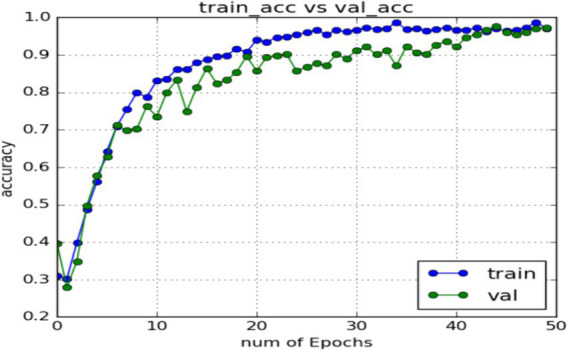
Accuracy of network in training set and test set.

**Figure 17:**
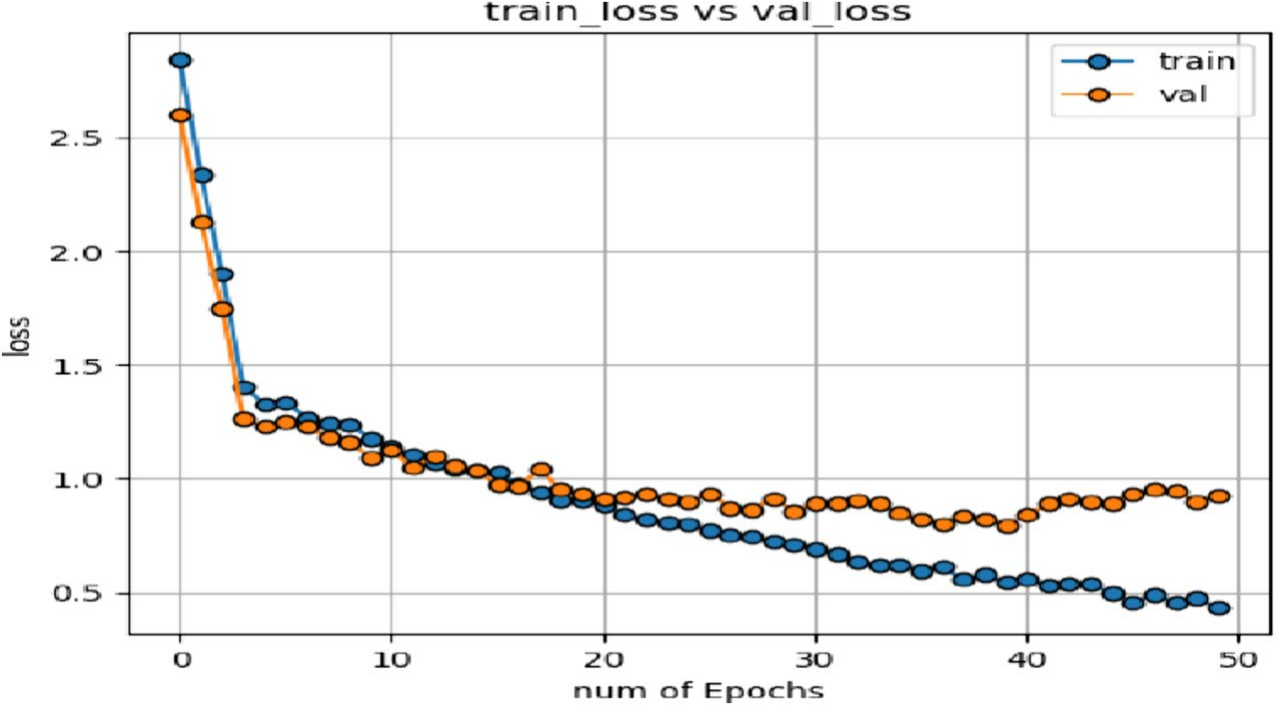
Loss function of network on training set and test set.

**Figure 18:**
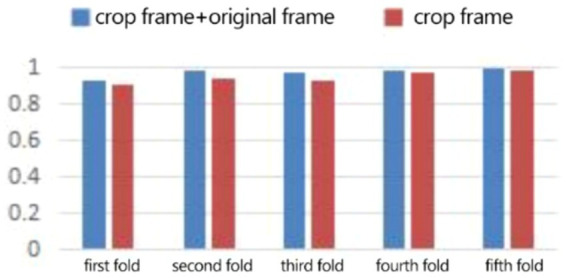
Compare the five-fold recognition rate of clip frames with clip frames + original frames.

**Figure 19:**
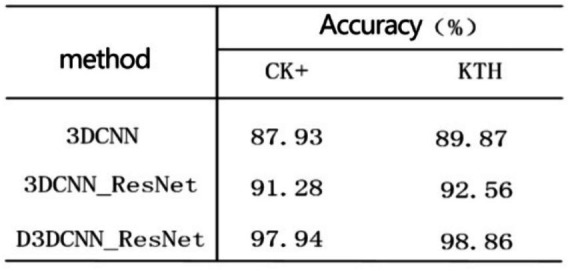
The average recognition rate (%) of clip frames and clip frames + original frames.

In order to verify the generalization of the D3DCNN_ResNet, it is also applied to behavior recognition. Figures 16, 17 show the iterative process of the network in the KTH data set. Through the recognition accuracy curve and loss function curve, it can be concluded that the method has good recognition performance, and the best recognition rate can be 98.86%. Figure 18 shows the 50% recognition accuracy of extracting features by using the crop frame alone, and fusing the crop frame and the original frame. Figure 19 shows the corresponding average recognition accuracy. It can be seen from the two figures that the recognition accuracy of the proposed method is increased by 2.52% with obvious effect.

Table 2 compares the D3DCNN_ResNet with the advanced methods that have been published on the KTH dataset. Jia et al. (2018) uses linear sequence difference analysis method to reduce the dimension of video data for human behavior recognition. Wang et al. (2011) classifies human behavior by using dense trajectory features. Tong et al. (2013) uses the dense block of three-dimensional residuals as the basic module of the network, and uses the local feature aggregation adaptive method to learn the local dense features of human behavior. Wang et al. (2013) is based on the MBH descriptor characterization feature of differential optical flow, and obtains a good recognition rate. Zhang et al. (2019) improves 3DCNN network by dividing 3DCNN into two convolution kernels in spatial domain and time domain to extract spatiotemporal features in parallel to improve model efficiency. Compared with the above methods, this method has achieved better recognition results, and the recognition rate has reached 98.86%, which is 3.26, 4.66, 5.36, and 3.56% higher than Jia et al. (2018), Wang et al. (2011), Tong et al., 2013, and Wang et al. (2013), respectively. Compared with Zhang et al. (2019), 3DCNN is also split. In this paper, two convolution kernels in spatial domain and time domain are connected in a serial way. The verification shows that it is better than the parallel way. The recognition rate of single crop frame is better than 0.14%, and the fusion recognition rate of crop frame and original frame is better than 2.66%.

**Table 2:** Comparison results of different algorithms on KTH.

Method	Feature	Recognition rate
Jia et al. (2018)	LSDA	95.6
Wang et al. (2011)	HOG-HOF-MBH	94.2
Tong et al. (2013)	3D-DenseNet	93.5
Wang et al. (2013)	Interest point detection based on flow vorticity	95.3
Zhang et al. (2019)	3DCNN	96.2
This article	D3DCNN ResNet	98.9

### 4.4 Performance evaluation of improved D3DCNN_ResNet

After the above experimental verification, the recognition rate is higher than that of extracting a single local feature of the crop frame by extracting the local feature of the crop frame in parallel and fusing the global feature of the original frame. In order to further verify the effectiveness of the proposed D3DCNN_ResNet, the D3DCNN network without residual connection and dense connection, the 3DCNN network with residual connection but without dense connection, and 3DCNN network without residual connection but with dense connection, are compared with D3DCNN_ResNet with residual connection and dense connection. As shown in Table 3, on CK+ dataset, the recognition rate of improved D3DCNN_ResNet is higher than that of 3DCNN and 3DCNN_ResNET by 10.01 and 6.66% respectively. On the KTH data set, the recognition rate is increased by 8.99 and 6.3% respectively. In conclusion, the D3DCNN_ResNet can effectively extract the feature information of video frames and improve the recognition accuracy.

**Table 3:** Accuracy of different algorithms on CK+ and KTH.

Method	Accuracy (%)	
	CK+	KTH
3DCNN	87.93	89.87
3DCNN_ResNet	91.28	92.56
D3DCNN_ResNet	97.94	98.86

## 5 Conclusion

This paper presents a new parallel D3DCNN_ResNet model structure. It divides the 3D convolutional neural network into two convolutions in spatial domain and time domain through the analysis of video sequence, extracts the spatiotemporal features in the video sequence in a serial manner, and adds a shortcut of residual network to increase the depth of the network. It solves the problem of excessive parameters and high computational costs in traditional 3D convolutional neural networks as the network depth increases. Moreover, dense connections are added in the residual block to fuse high-level and low-level features, maximizing the flow of feature information. The combination of residual blocks and dense connections not only reduces feature redundancy and improves the gradient correlation of the network, but also reduces the amount of network parameters, making the model have a certain anti over fitting effect. Through data preprocessing and data enhancement, the robustness and generalization of the model are enhanced, and it is not easy to be disturbed by external environmental factors. The clip frame and the original frame of the recognition region obtained from SSD target detection are trained respectively. Then multi-level features are extracted in parallel, and the classification is fused. Compared with a single network model, the parallel network can extract the local or even the overall spatial feature information of the image sequence more completely and effectively so as to improve the recognition rate. In the conventional teaching classroom, the students’ feedback on facial expression is an important way for teachers to know whether the student is suitable for his own teaching. However, the teacher will not always pay attention to the student’s expression and analyze of it, nor can he fully take into account the expression changes of all the students in the class. In this case, it is very meaningful to use computer technology as an assistant teacher to identify and record the expressions of students, analyze the quality of the class, and adjust the teaching progress to improve the teaching method. Through the application of the D3DCNN_ResNet, it is of great significance to effectively improve the recognition rate of classroom expressions. At the same time, the traditional English classroom oral speech training purely relies on speech recognition, which has a flaw that it is impossible to maintain a high recognition accuracy in a noisy environment. The visual recognition method is not affected by the ambient sound, and the accurate path of speech recognition can be improved through multimodal recognition. The new model structure of the D3DCNN_ResNet mentioned in this paper can also assist in speech interaction and image recognition, which is widely used in the field of English spoken speech teaching. However, given the complexity of the language environment, it will take time to truly put into practice, and it is still necessary to further strengthen the integration of research in areas such as big data visual analysis and artificial intelligence technology.

## Data Availability

The original contributions presented in the study are included in the article/supplementary material, further inquiries can be directed to the corresponding author.
